# The morphogenesis of the rabbit meibomian gland in relation to sex hormones: Immunohistochemical and transmission electron microscopy studies

**DOI:** 10.1186/s40850-022-00149-2

**Published:** 2022-08-11

**Authors:** Sara M. M. El-Desoky, Nada Abdellah

**Affiliations:** 1 Department of Anatomy and Embryology, Faculty of Veterinary Medicine, Assiut University, Assiut, 71526 Egypt; 2 Department of Histology, Faculty of Veterinary Medicine, Sohag University, Sohag, 82524 Egypt

**Keywords:** Meibocyte, Androgen, Estrogen, Cytokeratin14, Stem cells

## Abstract

Rabbits have been proposed as a model for the human meibomian gland (MG), a large specific sebaceous gland in the eyelid that consists of secretory acini arranged laterally and related to the central duct via short ductules, with the central duct continuing as an excretory duct to open at the free margin of the lid. First detected at embryonic day 18 as an aggregation of mesenchymal cells in the tarsal plate, it completes its development approximately 2 weeks postnatal when the separation of the eyelids is completed. The Transmission electron microscopy supports the meibocytes’ gradient maturation to the meibum’s synthesis. While the differentiating cells, their cytoplasm, are well packed with lipid droplets, the basal cells are characterized by a high nuclear to cytoplasm ratio. The androgen and estrogen receptor proteins are expressed in the basal cell and the meibocytes, and increase in age increases in the expression of these proteins. Additionally, the cytokeratin (CK14) is expressed in the basal and differentiating cells of the acini and the ductal epithelium. Therefore, the duct cells of the MG are common in all stem cells. These data concluded that the MG plays a major role in maintaining the health of the ocular surface and preservation of visual acuity. Any abnormalities in the structure of the MG lead to its dysfunction and changes in lipid secretion.

## Introduction

It is challenging to identify the experimental animal models for human craniofacial development, growth, and dysmorphology. The rabbit is a suitable experimental model because it is an excellent model for phylogenetically disparate [1], not entirely hinging on phylogenetic affinity.

The meibomian gland (MG) plays a major role in health maintenance, the ocular surface integrity, and the preservation of visual acuity [2]. This produces the lipid, which forms the superficial layer of the tear film, which consists of three layers. The first layer is the oily layer produced by the MG and zeis gland. The second layer is aqueous and secreted from the lacrimal, nictitans, and accessory (Krause’s and Wolfring’s) glands. The third layer is the mucous layer that forms from the goblet cells of the conjunctiva mainly and the third eyelid gland [3]. Therefore, the lipid produced from the MG protects the tear film from aqueous evaporation and stabilizes the tear film by decreasing the surface tension [4].

The MG is one of the important secretory glands and is considered a large specific sebaceous gland in the eyelid. That is arranged in the tarsal plate of the eyelids [5]. The primordium of the MG begins as an aggregation of the ectodermal epithelium. Then, the epithelial cells invaginate into the mesenchyme, differentiate, branch, and develop into the mature MG around 2 weeks postnatal concurrent with eyes opening [6]. Many congenital anomalies of the eyelids as a result of abnormal proliferation of tissue, fusion, and reseparation of the eyelid [7].

The meibum secreted by the MG protects the eye from microbial invasion, assisting in tight closure of the lid margin during sleep and regulating the tension force of the tear film [8]. Therefore, MG dysfunction is characterized by chronic abnormalities of the structure of the MG and changes in lipid secretion. Most MG dysfunction leads to dry eye diseases [9, 10]. However, many therapies are experimented to treat dry eye symptoms as androgen replacement therapy. Therefore, through the understanding of sex action hormones on the MG, it was found that the androgen and estrogen hormones regulate their function, which androgen support their function, and estrogen act antagonistically [11, 12].

Keratin proteins form the cytoskeletal and structural components of the epithelial cells. The keratin proteins are important for tissue stability, cell morphology maintenance, cell-to-cell communication, and cell proliferation [13]. Additionally, the keratin expression is considered specific for tissue, differentiation, and development regulation as CK14 keratin is present in the basal cells of the stratified epithelium [14].

This study is aimed at providing a basic understanding of the developmental and morphogenesis of the structures of the rabbit MG at the level of light and transmission electron microscopy and understanding the foundation of congenital anomalies origins. As well as identifying the expression of the sex hormones in their tissues and the role of these hormones in regulating the MG function.

## Materials and methods

### Sample collection

This study was conducted on 28 white New Zealand rabbits at pre-and postnatal life; four rabbits at every age. These rabbits were obtained from the farm of the Faculty of Agriculture, Assiut University, Egypt. The mating day was considered the first day of embryonic life. The pregnant female rabbits were collected at different stages of pregnancy, beginning from the embryonic day (ED)18, 20, 25, and 30 days by which gestation occurred. The postnatal rabbits were collected from the males at the ages of one and 2 weeks as well as 1 month, in which the rabbit eyes were completely opened, and the eyes were functionally active.

### Tissue preparing

After euthanasia, the rabbit was sedated by anesthesia using sevoflurane (United States Pharmacopeia (USP)) as an inhalational agent to loss consciousness and then slaughtered in the cervical region following euthanasia techniques. In the case of fetuses, the rabbit heads were obtained, while the whole eyes and its eyelids were collected in the postnatal ones and were fixed immediately in 10% neutral buffered formalin. Then, the specimens were processed for the paraffin histology by dehydrated in ascending grades of alcohol, cleared in xylene, and impregnated with melted paraffin wax. Finally, the paraffin blocks were cut into thin sections (3 μm thick) using Richert Leica RM 2125 Microtome and mounted on glass slides. Then, the sections were stained with Harris’s hematoxylin and eosin, Crossmon’s Trichrome, and Picro Sirius red method to stain the collagen fibers with red and the muscle fibers and cytoplasm with yellow [15].

### Semithin sections and transmission Electron microscopy preparations

Small pieces 2.0–3.0 mm long from the specimens were placed on 2.5% cold glutaraldehyde in phosphate buffer (pH 7.2) for 24 h. The pieces were washed twice in 0.1-M phosphate buffer and then post-fixed in 1% osmium tetraoxide, in the same buffer. The post-fixed pieces were dehydrated in graded alcohols and embedded in Araldite resin. Semithin sections (1 μm) in thickness will be stained with 1% toluidine blue.

Specimens from the upper eyelids of three rabbits at one, 2 weeks, and 1 month postnatal were used for semithin sections, which were fixed in 2.5% cold glutaraldehyde in phosphate buffer (pH 7.2) for 24 h. The pieces were washed twice in 0.1-M phosphate buffer and then post-fixed in 1% osmium tetraoxide, in the same buffer. The post-fixed pieces were dehydrated in graded alcohols and embedded in Araldite resin. Semithin sections were cut at (1 μm) in thickness and stained with 1% toluidine blue [16]. Ultra-thin sections obtained from a Reichert ultra-microtome were stained with uranyl acetate and lead citrate [17]. Transmission electron microscopy (TEM) images were captured by the JEOL-100CXII electron microscope (JEOL, Tokyo, Japan) at the Electron Microscopy Unit of Assiut University.

### Immunohistochemistry

Immunohistochemistry staining has been performed on the paraffin blocks as mentioned by [18]. First, sections were dewaxed by xylene, rehydrated by descending grade of ethanol, and rinsed thrice with 0.1-M PBS for 10 min. Then, the antigen retrieval was conducted using 0.1-M sodium citrate buffer solution at (pH 6) for 5 min using a microwave (600 w). After that, the sections were cooled at room temperature for 20 min and rinsed with PBS (pH 7.4) for 10 min.

The endogenous peroxidase activity was inhibited by 3% H_2_O_2_ in H_2_O for 30 min at room temperature, and the sections were washed thrice using PBS for 5 min. The sections were incubated in the blocking solution formed by 10% normal donkey serum + 0.2% Triton-X100/PBS for 2 h at room temperature. Subsequently, sections were incubated overnight at 4 °C with the following antibodies: mouse monoclonal androgen receptors (1:200, Santa Cruz biotechnology 441, Catalog No. Sc-7305), rabbit polyclonal estrogen receptors (1:200, Thermo Fisher scientific, Catalog No. RM9101-S0), and mouse monoclonal anti-cytokeratin 14 (1:500, Santa Cruz biotechnology, Catalog No. sc-53,253). Then, the sections were rinsed thrice for 10 min in 0.2% Triton-X 100/PBS and incubated with biotinylated secondary antibody goat anti-rabbit IgG, and donkey anti-mouse IgG (from Dako, Hamburg, Germany) diluted at 1:200 for 2 h at room temperature. Then, the sections were incubated with vectastain ABC (Avidin-Biotin complex) reagent for 45 min at room temperature. Visualization of the reaction was conducted with 3,3′-Diaminobenzidine (DAB) for 5–10 min. Next, the sections were counterstained with Harris hematoxylin for 30 s. Then, the sections were dehydrated by ascending grade of ethanol, cleared by xylene, and covered by DPX (Dibutyl phthalate Polystyrene Xylene). Immunohistochemical staining was evaluated using a Leitz Dialux 20 Microscope and photographed by a Canon digital camera (Canon Powershot A95).

## Results

**Fig. 1:**
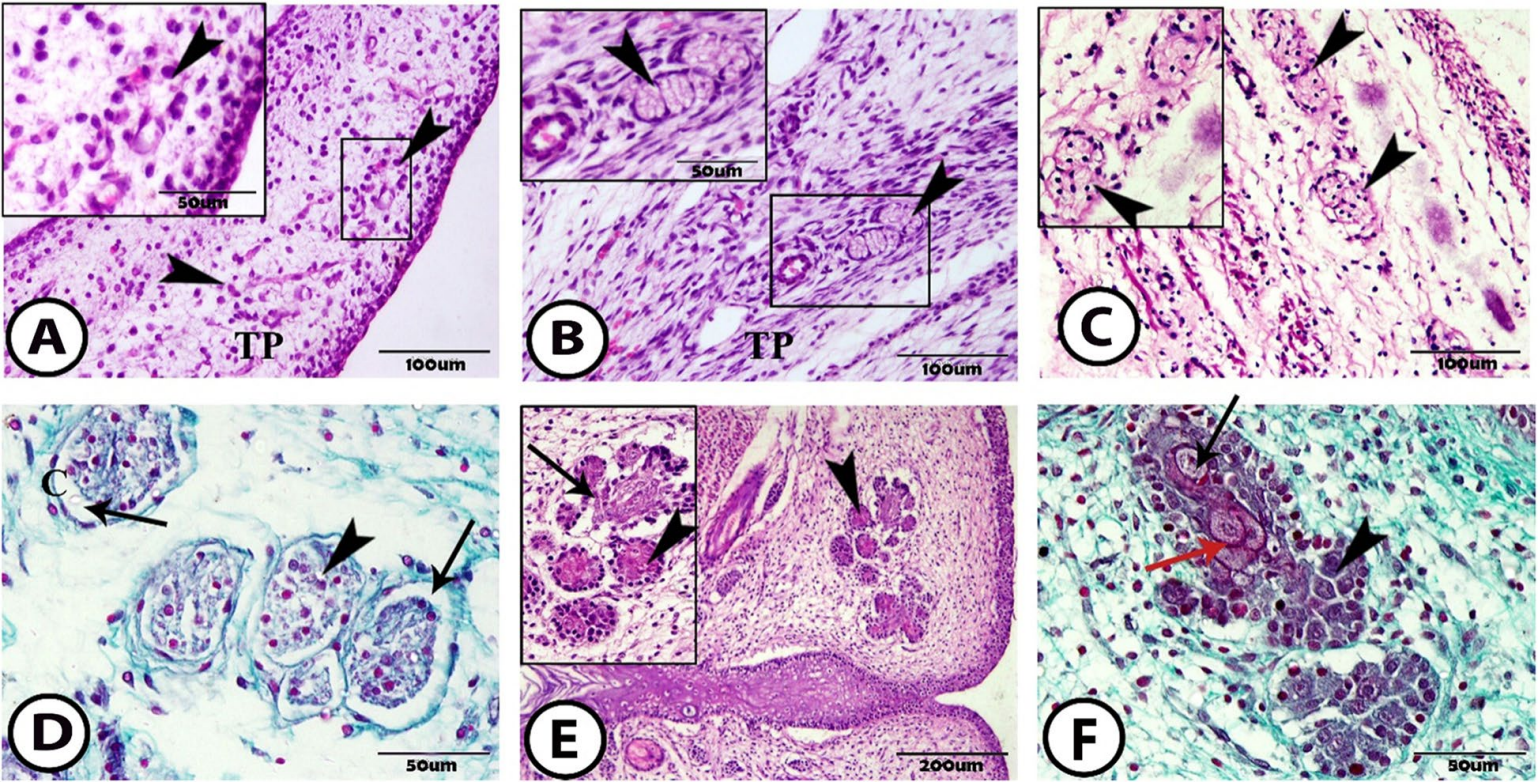
The prenatal development of the meibomian gland **A** ED18 shows the aggregation of the mesenchymal cells (arrowheads) in the mesenchymal condensation of the tarsal plate (TP) (Hematoxylin and Eosin (H&E)). **B** ED20 showing the MG anlage consists of the solid masses of cells (arrowheads) invaginated in the tarsal plate (TP) (H&E). **C** ED25 shows the epithelial cells aggregation of the MG anlages (arrowheads) in the tarsal plate (TP) (H&E). **D** ED25 shows a cross-section in the MG anlages, which consists of an epithelial core (arrowhead) surrounded by a capsule (C) and a hollow space (arrows) between them (Crossmon’s Trichrome). **E** & **F** ED30 shows branching MG, which composes of lateral outgrowth acini (arrowheads) and connecting ductules (arrows) which contains keratohyalin granules (red arrow). (H&E and Crossmon’s Trichrome respectively)

The MG anlage was not observed until embryonic day ED 18. On this day, some mesenchymal cells are arranged circularly at the underlying mesenchymal condensation of the tarsal plate. These cells represented the anlage of the MG (Fig. 1A).

At ED 20, the fusion of the upper and lower eyelids was completed. The epithelium of the MG anlage formed the solid masses of cells, which were invaginated into the fibrous tissue of the tarsal plate near the ocular surface of the eyelid margin (Fig. 1B).

At ED 25, the MG anlages were now observed as a well-defined structure. These MG anlages became larger and extended longer and deeper in the tarsal plate (Fig. 1C). In the cross-section, the MG anlages appeared as the irregular aggregation of the epithelial core with the defined nucleus and surrounded by the connective tissue capsule. Additionally, a hollow space was observed between this epithelial core and its capsule (Fig. 1D).

At ED 30, the full semester of pregnancy in rabbits, the MG began to branch and formed the lateral outgrowths from the epithelial core that became differentiated into secretory acini and connecting ductules. The epithelial core was differentiated into a single layer of small basal cells with a dense nucleus, which were located at the periphery, and meibocytes that were cuboidal-like secreting epithelium with a rounded nucleus and dense cytoplasm. Keratohyalin granules were observed in the luminal epithelium of the MG (Fig. 1E, F).

**Fig. 2:**
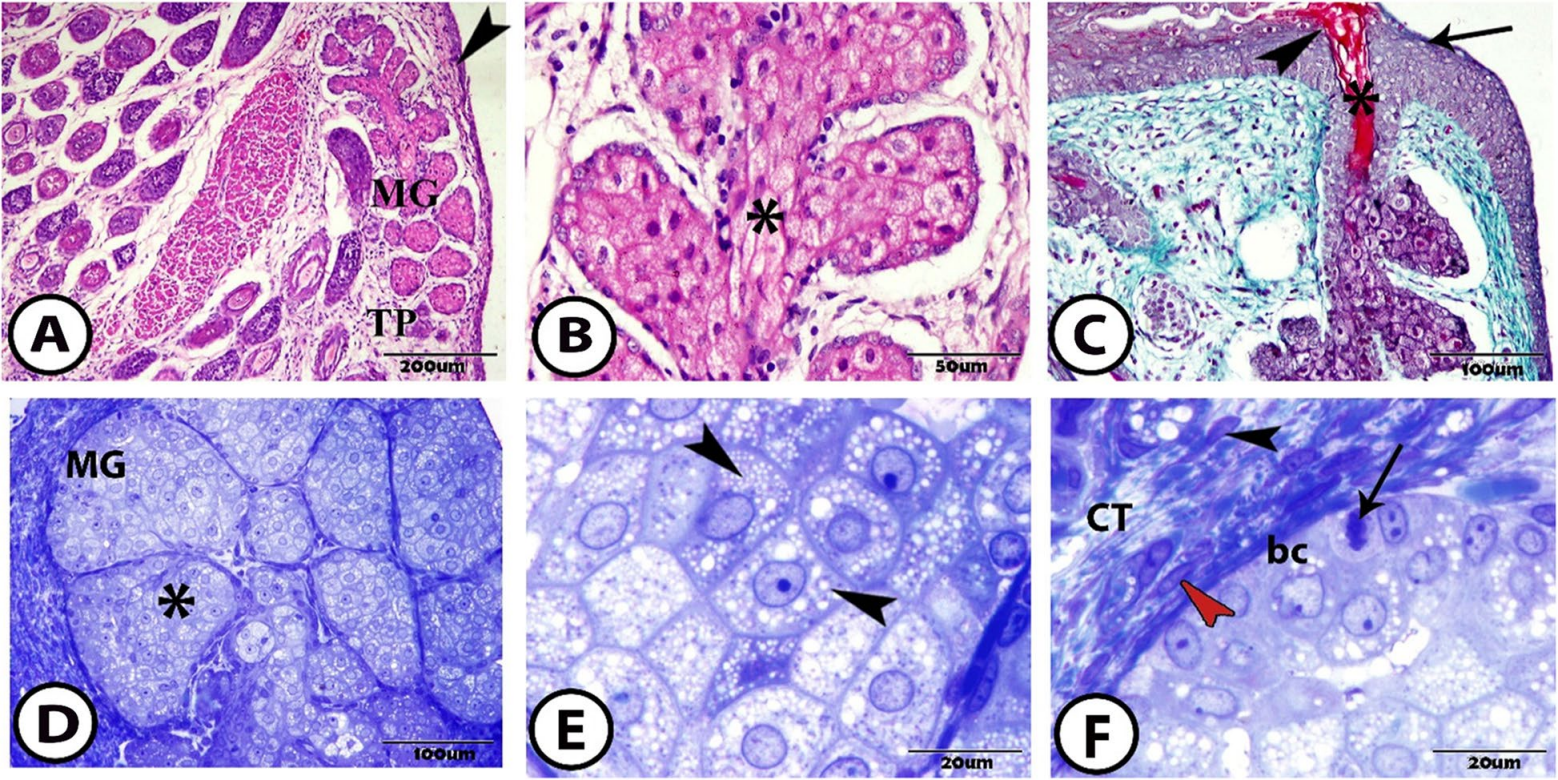
The meibomian gland at one-week postnatal **A** meibomian gland (MG) extends in the tarsal plate (TP) along the posterior border of the eyelid (arrowhead) (Hematoxylin and Eosin (H&E)). **B** The central duct of the MG (asterisk) obstructs with multilayered squamous epithelium (H&E). **C** The MG opens at the orifice (arrowhead) onto the free margin of the lid (arrow) close to the posterior border of the eyelids. The keratin lamellae (asterisk) are present at the terminal part of the central duct (Crossmon’s Trichrome). **D** Semithin section in the acini of the MG that filled with meibocytes (asterisk) (toluidine blue). **E** Semithin section in the meibocytes that accumulate of the lipid vacuoles inside it and appear as foamy cytoplasm (arrowheads) (toluidine blue). **F** The semithin section in the acini shows that the basal cells (bc) appear signs of mitosis (arrow). The connective tissue (CT) between the cells contains fibroblasts (black arrowhead) and myoblasts (red arrowhead) (toluidine blue)

At 1 week after birth, the MG became longer and extended deeper in the tarsal plate and occupied two-thirds of the length of the plate (Fig. 2A). In this stage, the MG was composed of secretory acini that connected to the central duct through the connecting ductules. The central duct in this stage was obstructed with multilayered squamous epithelium (Fig. 2B). Then, the central duct opened onto the free margin of the lid close to the posterior border of the eyelids. The terminal part of the central duct was covered by the keratin lamellae that were growing from the surface of the free margin of the lid (Fig. 2C).

Secretory acini are a special type of sebaceous gland. These acini were occupied with secretory cells called meibocytes (Fig. 2D). Inside these cells was observed a different degree of accumulation of lipid droplets in their cytoplasm and appeared as a foamy cytoplasm with a central rounded nucleus (Fig. 2E). Most periphery cells were the basal cells that appeared to have signs of mitosis. The basal acinar cells were located in the periphery and appeared darker and smaller than the meibocytes with a central oval nucleus. The connective tissue capsule surrounded the MG. The abundant connective tissue fibers were observed between the acini, and then the connective tissue extended between their meibocytes, and the myoblast was presented between the cells (Fig. 2F).

**Fig. 3:**
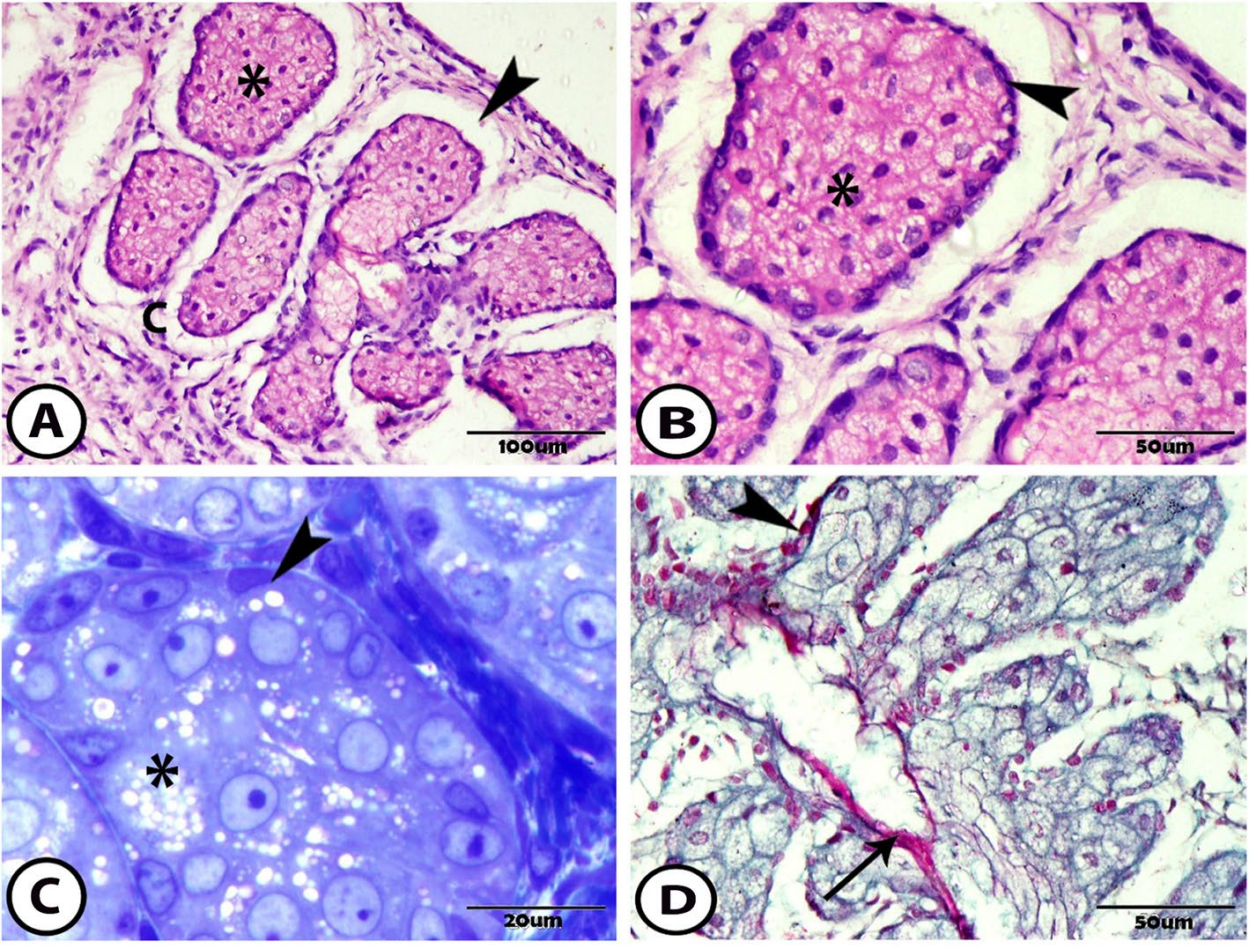
The meibomian gland at two-weeks postnatal **A** General view of the MG showing its acini (asterisk) that is surrounded by the capsule (**C**) and separated from it by a hollow space (arrowhead) (Hematoxylin and Eosin (H&E)). **B** & **C** The acini of the MG is composed of meibocytes with foamy cytoplasm (asterisks) and darker periphery located basal cells (arrowheads) (H&E and toluidine blue respectively). **D** The connecting ductules are lined by cuboidal epithelium (arrowhead). The central duct is opened and lined by a keratinizing layer (arrow) (Crossmon’s Trichrome)

At 2 weeks after birth, the separation of the upper and lower eyelids was completed. The MG in this stage branched more, looking like a grape attached to the central stalk. They extended deeper into the tarsal plate (Fig. 3A). In the cross-section, the acini were composed of abundant meibocytes with foamy cytoplasm and darker basal cells (Fig. 3B, C).

These acini connected with small ductules to the large central duct. These connecting ductules were lined by cuboidal epithelium. These epithelial cells contained keratohyalin granules. The central epithelial cells were apoptosis and formed the central duct. The central duct was lined by a keratinizing layer composed of numerous keratohyalin granules (Fig. 3D).

**Fig. 4:**
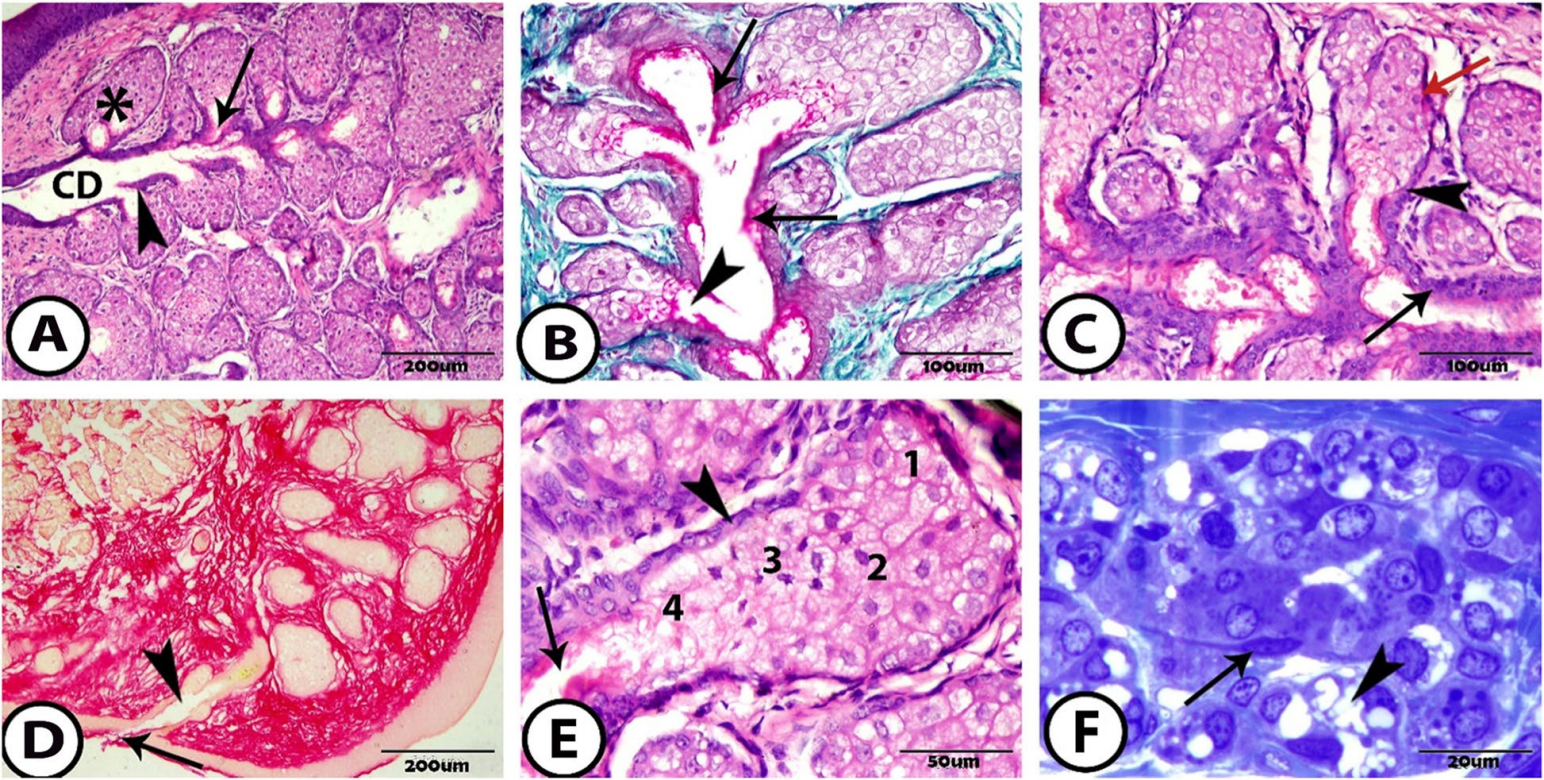
The meibomian gland at one month postnatal **A** General view of the well-developed MG showing its acini (asterisk), connecting ductules (arrow) and central duct (CD) that the sharp spurs (arrowhead) are found at the entrance of the connecting ductules to the central duct (Hematoxylin and Eosin (H&E)). **B** The single ductules are connected with one or more acini (arrowhead). The connecting ductules and central duct are lined by keratinizing layer (arrows) (Crossmon’s Trichrome). **C** A sharp transition is observed from the basal cells (red arrow) to the cuboidal cells of the connecting ductules (arrowhead). The central duct is lined by 2 to 3 layers of cuboidal cells (arrow) (H&E). **D** The excretory duct forms the ampulla (arrowhead) before opening by its orifice at the free margin of the eyelid (arrow) (Picro Sirius red). **E** Showing the maturation of the meibocytes from basal cell (arrowhead), 1. differentiating cell, 2. mature cell, 3. Hyper-mature cells that appear shrinkage with a pyknotic nucleus, 4. The cells near the connecting ductules (arrow) are filled with the secretory meibum (H&E). **F** The semithin section in the acini shows the meibocytes filled with numerous lipid vacuoles (arrowhead) and the myoblast presents between the cells (arrow) (toluidine blue)

At 1 month, the MG reached a fully developed in appearance, extended in length, and occupied almost the entire length of the tarsal plate. In this stage, the duct system of MG became clearer and was composed of small connecting ductules, which opened in a large straight central duct and were continuous as an excretory duct opening at the free margin of the lid close to the posterior and ocular border of the eyelids (Fig. 4A).

The connecting ductules were small, short, and lateral in position. Sometimes the single ductules connected with one or more secretory acini (Fig. 4B). These ductules were lined by one layer of the cuboidal cells. At the junction between the acini and ductules, a sharp transition was observed from the basal cells of the acini to the cuboidal cells of the ductules (Fig. 4C). These ductules were lined by a keratinizing layer of keratohyalin granules (Fig. 4B).

The connecting ductules obliquely opened in the central duct. At the entrance into the central duct, sharp spurs were formed that were composed of epithelial cells (Fig. 4A). The central duct was large, long, and straight with a wide lumen. It was lined through two to three-layered cuboidal cells. The terminal part of the central duct that continues as the excretory duct was dilated and formed the ampulla that stored the secreting meibum (Fig. 4D).

The secretory acini of the MG were located laterally and had a holocrine mode of secretion. The basal cells were located at the peripheral margins. The outer meibocytes were the differentiating ones that produced the lipid droplets and accumulated as a vacuole in the cytoplasm, giving it this foamy appearance and containing a rounded nucleus.

The meibocytes then became more mature with the intact nucleus. Finally, the most central meibocytes (hyper-mature) were shrinkage, the nucleus became pyknotic, and the cell membrane disintegrated, and the whole cell formed the secretion, which was called the meibum (Fig. 4E). The lipid granules appeared numerous and small in the differentiating cells then fused with each other and became larger in size in the central mature cells. At this age, the connective tissue between the meibocytes disappeared but was still present between the acini, while the meibocytes became fully developed and the myoblast was present between the meibocytes (Fig. 4F).

### Transmission electron microscopy

**Fig. 5:**
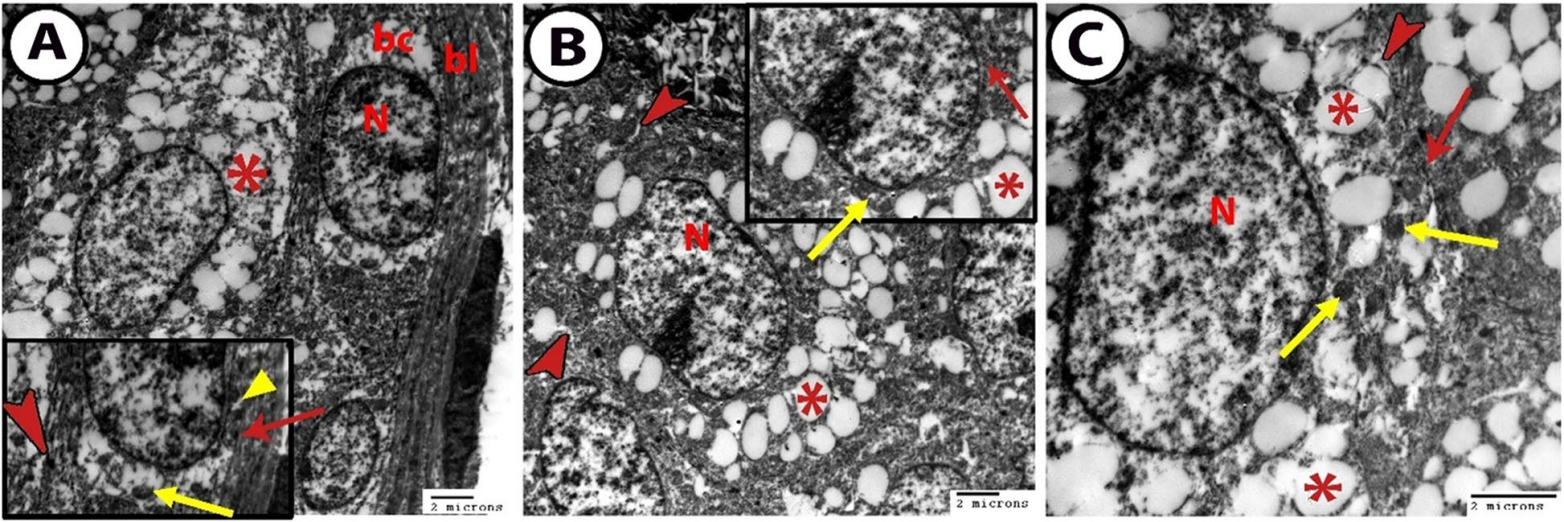
TEM of the meibomian gland showing **A** The basal cell (bc) links the basal lamina (bl) by hemidesmosomes (red arrow) and the differentiating meibocytes (asterisk) by desmosomes (red arrowhead). It contains a heterochromatin nucleus (N), mitochondria (yellow arrow), and smooth endoplasmic reticulum (yellow arrowhead) in the cytoplasm. **B** The differentiating cell contains a nucleus (N) with irregular cluster chromatin and the cytoplasm with the rough endoplasmic reticulum (arrow), smooth endoplasmic reticulum (arrowheads), mitochondria (yellow arrow), and different sizes of lipid droplets (asterisks). **C** The mature cell showing the central nucleus (N) and the cytoplasm contains large lipid droplets (asterisks), numerous mitochondria (yellow arrows), rough endoplasmic reticulum (red arrow), and smooth endoplasmic reticulum (arrowhead)

The transmission electron microscopy (TEM) of the acinar cells of the MG varied according to the differentiating stage of the holocrine secretion. First, the basal cells were situated at the periphery of the acinus. Then, the differentiating meibocytes were presented, and the mature cells occupied the center of the acinus. The basal cells were located between the basal lamina and the differentiating meibocytes. These cells were linked with the basal lamina by hemidesmosomes. These basal cells were small with a large heterochromatin nucleus and a defined nucleolus. The heterochromatin was lined along the nuclear membrane. The cytoplasm was scanty and contained numerous mitochondria, rough endoplasmic reticulum, and smooth endoplasmic reticulum (Fig. 5A).

The differentiating meibocytes were joined with the neighboring basal cells by desmosome. Their nuclei were large and occupied the center of the cells with the defined nucleolus. The chromatin clustered irregularly. Lipogenesis was stimulated, and the lipid droplets accelerated in the cytoplasm around the nucleus. Therefore, the cytoplasm was well packed by the lipid droplets and smooth endoplasmic reticulum with few profiles of the rough endoplasmic reticulum. Mitochondria with different shapes and sizes were scattered in the cytoplasm (Fig. 5B).

In the mature cells, the lipid droplets fused and became larger in size and occupied a large proportion of the cytoplasm. Also, the other organelles as smooth endoplasmic reticulum, rough endoplasmic reticulum, and different shapes and sizes of mitochondria were observed in the cytoplasm (Fig. 5C).

### Immunohistochemistry

**Fig. 6:**
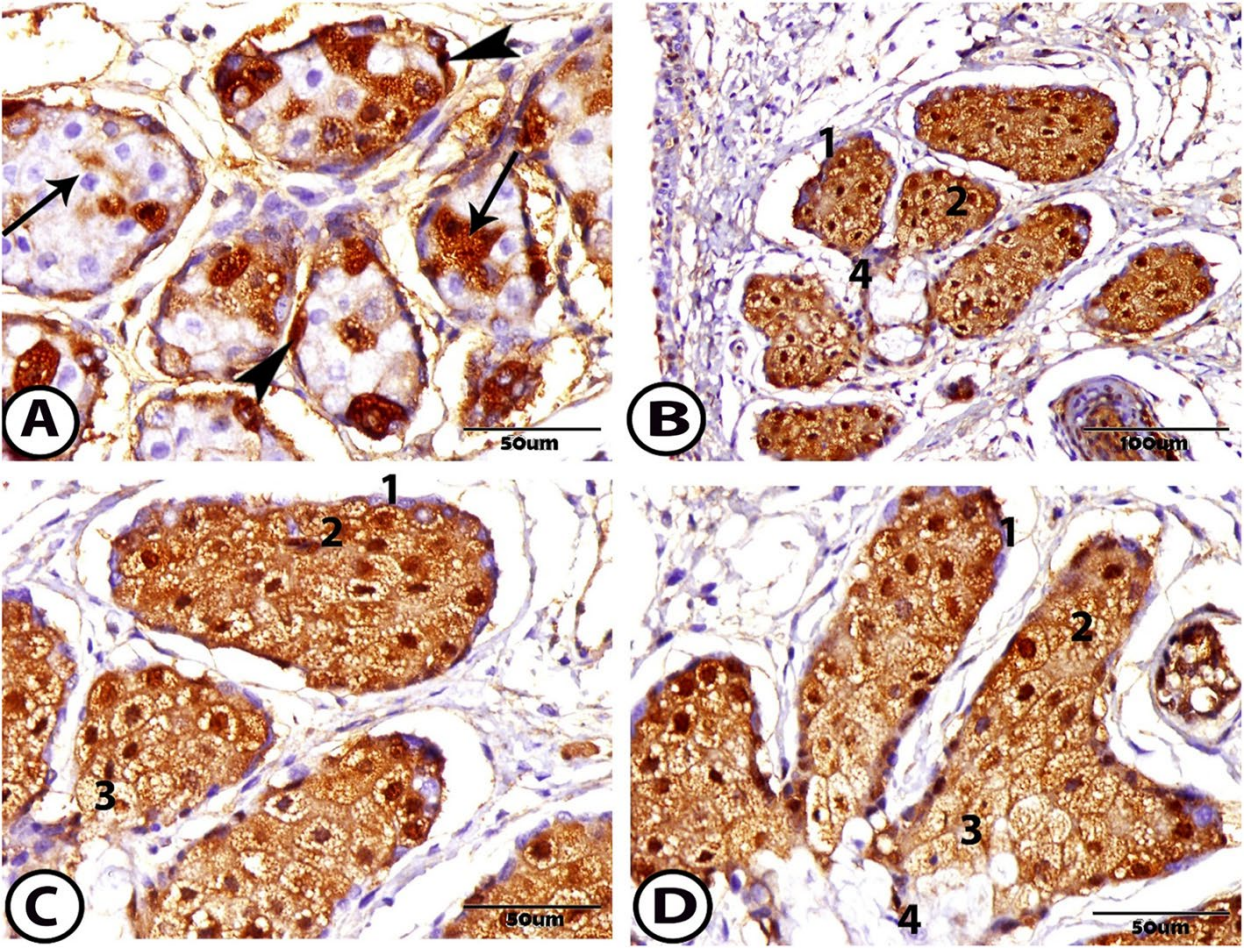
Immunohistochemical staining of the meibomian gland to androgen protein **A** At one-week postnatal showing positive expression to the basal cells (arrowheads) and different immunoreactivity to the differentiating cells (arrows). **B-D** At one month postnatal showing the different immunoreactivity to the 1. basal cell (not reacted nucleus), 2. differentiating cell (positive), 3. mature cell (positive), and 4. ductal cell (positive)

Expression of the androgen receptor protein varied according to age. At 1 week, the androgen receptor protein appeared in some of the acinar epithelial cells with positive acinar nuclei, while other cells did not react with androgen receptors. Most of the basal cells with their nuclei showed a positive reaction to the androgen receptor (Fig. 6A). Alternatively, at 1 month, the androgen receptor protein was presented exclusively within the epithelial cells of the acini and its nuclei. The mature cells in the center of the acini and near the duct showed positive expression of the androgen protein, while this reaction did not appear in the nuclei of the basal cells. Also, androgen receptor protein was expressed in the epithelial cells of the connecting ductules and central duct (Fig. 6B–D).

**Fig. 7:**
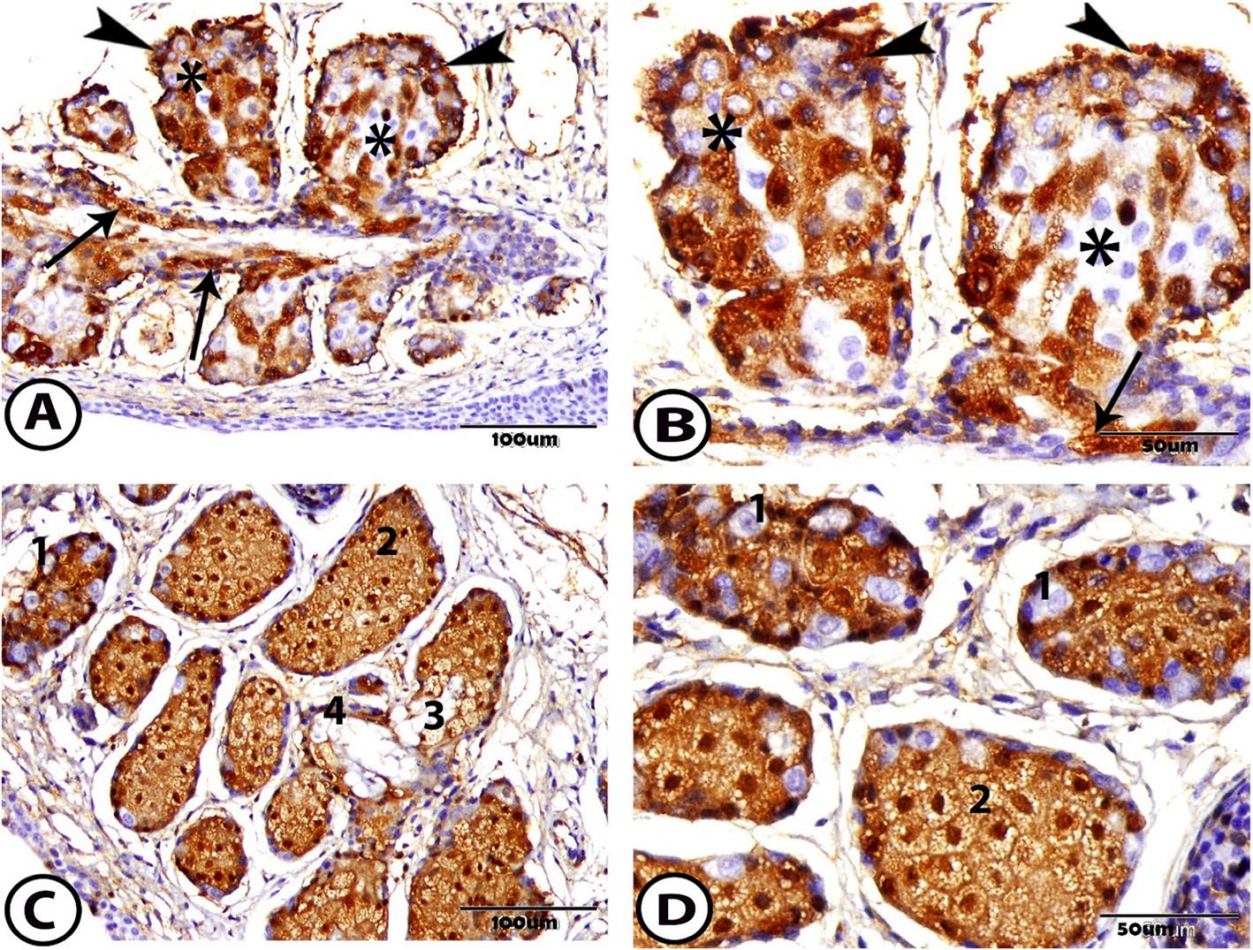
Immunohistochemical staining of the meibomian gland to estrogen protein **A** & **B** At one-week postnatal showing the different immunoreactivity to the acinar cells (asterisks), positive reaction to the basal cell (arrowheads), and ductal epithelial cells (arrows). **C** & **D** At one month postnatal showing the different immunoreactivity to the 1. basal cell (many nuclei not reacted), 2. differentiating cell (positive), 3. mature cell (not reacted), and 4. ductal cell (positive)

In this research, at 1 week, the antibodies for estrogen receptors were expressed within the basal cells and their nuclei. This estrogen reaction appeared in varying degrees of expression inside the acini of the MG some meibocytes gave a positive reaction and other cells had a negative reaction to an estrogen receptor protein. Also, the estrogen receptor protein appeared to have a positive expression in the ductal epithelial cells (Fig. 7A, B). Besides it, at 1 month, the expression of estrogen receptor protein was cleared, that it was exclusively located within the acinar cells with their nuclei, except that a few cells did not respond to this reaction. In contrast, many of the nuclei of the basal cells had not reacted with estrogen receptors other than the previous age. The mature cells near the duct did not react to the estrogen protein. However, the ductal epithelial cells still reacted positively to the estrogen receptor protein (Fig. 7C, D).

**Fig. 8:**
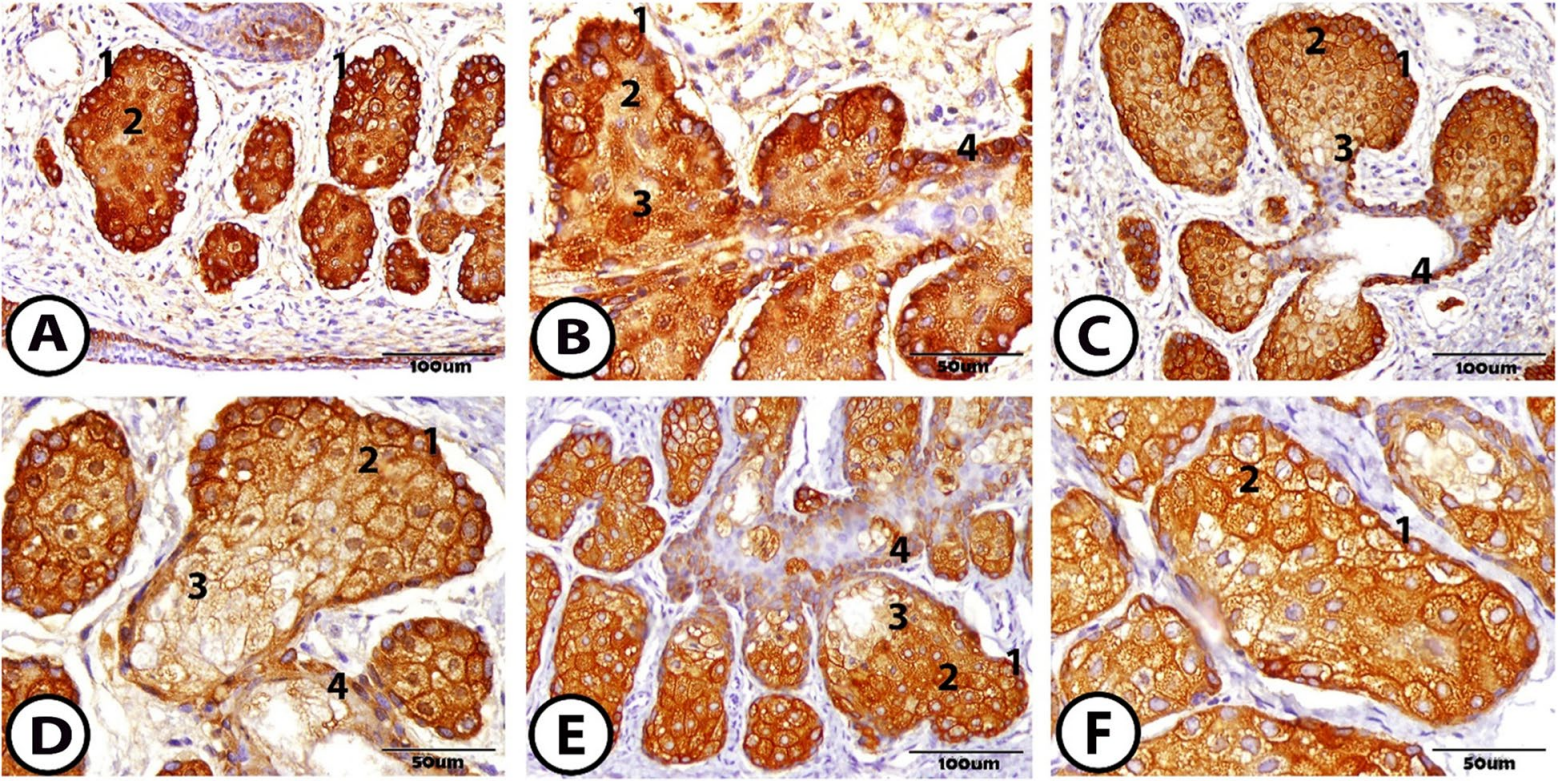
Immunohistochemical staining of the meibomian gland to CK14 **A** & **B** At one-week postnatal, **C** & **D** At two-weeks postnatal, and **E** & **F** At one-month postnatal showing that the whole acini give a positive reaction to CK14 from 1. basal cell, 2. differentiating cell, 3. mature cell, and 4. ductal cell

Expression of CK14 to the MG appeared positively and clearly. At 1 week, the whole acini showed a positive reaction, the outer basal cells gave a positive reaction to CK14, and the differentiating cells showed wide immunoreactivity. Also, the inner mature cells reacted positively (Fig. 8A, B). At 2 weeks, the expression of CK14 also showed a positive reaction in the whole acini with a high reaction in the basal and differentiating cells. Furthermore, the epithelial cell layer of the ductules and the central duct showed a positive reaction to CK14 (Fig. 8C, D). Also, at 1 month, different immunoreactivity to CK14 was identified within the basal cells and the meibocytes (Fig. 8E, F).

## Discussion

This research focuses on describing the development of the MG in rabbits and explaining the role of the sex hormones in regulating its functions. The fusion of the fold of the eyelids is essential for differentiating the structure of the eyelid and the proper development of the MG. The failure in the closure of the eyelids is accompanied by a defect in the development of MG, which leads to failure to form the MG placode and does not occur in the invagination of the tarsal plate and epithelial cord branching indictable or few acinar regions [19].

The MG appeared as mesenchymal cells at ED18 and begin to develop into the solid masses of cells and invaginated in the tarsal plate by ED20. This started developing when the upper and lower eyelids are completely fused, which occurs at ED20 in rabbits but at 8 weeks [20] or 9 weeks in humans [21]. This means that in humans, the process of closure of the eyelids occurs in the first semester of the pregnancy (8–12 weeks), while in rabbits, it occurs at the end of the second semester of pregnancy (ED20). Therefore, the development of the MG in rabbits continues after birth until eyelid’s separation occurs, which is observed 2 weeks after birth; this result is similar with [19]. This explains why the rabbit is born with closed eyes. The structures are not fully developed at birth, and when they become fully developed and start functioning, the eyelids separate, while in humans, MG growth occurs from the third to the seventh month of gestation [22].

The eyelids structure development occurs according to the fusion of the lids, which leads to the amniotic fluid to play a role in this development. Firstly, the amniotic fluid contains a high concentration of sodium chloride (8 weeks) in humans. However, when the kidney begins to function, the uric acid, urea, and creatinine contents increase in the amniotic fluid, while the concentration of the sodium chloride decreases at 12 weeks, so the lids fuse at 8 weeks before the beginning of the renal function [20].

The MG anlage at ED25 appears as the irregular aggregation of the epithelial core and at the full semester of pregnancy in rabbits (ED30), the MG branches and differentiates into the secretory acini and connecting ductules. This first appearance of anlages of MG is also observed in humans around week 11 [23]. Furthermore, keratohyalin granules are observed at Ed30 in rabbits in the ductal epithelium of the MG. Also, this is observed in mice’s embryological life in the luminal epithelium, which explains why the hyperkeratinization of MG is a typical disease in adults [23].

At 1 week after birth, the MG is composed of secretory acini and a central duct obstructed by the epithelial cells. At 2 weeks, the separation of the upper and lower eyelids is completed, the central duct is opened, and the central epithelial cells are apoptosis. Therefore, when the eyelid is opened at 2 weeks, the central duct is formed. The acini of MG are formed at 7 months in humans when the eyelids are separated. There is no lytic substance in the secretion of the MG, which helps in the lid separation. In contrast, the oily consistency of the secretion helps in maintaining the eyelid separation [20].

In this study, the epithelial cells of the connecting ductules contain keratohyalin granules, and the central duct is lined by a keratinizing layer of keratohyalin granules. In contrast, [24], rats stated that the central duct is lined by stratified squamous epithelium and appears keratinized in some areas of the duct [25] stated that the terminal part of the central duct is lined by squamous epithelium with modified keratinization. It is thought that an increase in the amount of keratinization compared lipid production helps in separating the eyelids [26].

At 1 month, the MG becomes well developed and is considered a modified sebaceous gland but does not communicate with the hair follicles [27]. The multiple secretory acini arrange laterally and are related to the central duct via short ductules. The connecting ductules and the central duct are lined by one to three layers of cuboidal cells, respectively. In constrast, in rats, these ductules and ducts are lined by four layers of stratified squamous epithelium, and the excretory duct is lined by modified keratinized squamous epithelium [25]. In this study on rabbits, a sharp transition of the cells from the basal cells of the acini to the cuboidal cells of the connecting ductules is observed, which agrees with [28] in the monkey. In humans, this transition in cells is gradually from the basal meibocytes to the multilayered ductal epithelium that are longer in height [22]. Also, the meibocytes contain lipid droplets, while the ductules do not contain lipid droplets, but instead, contains keratohyalin granules in these cells [22].

Observations revealed the presence of myoblasts among the meibocytes and the central duct. These myoblast cells play a role in the release and transportation of the MG secretion to the lid orifice. While [29] suggested that there are striated fibers of Riolan’s muscle around the terminal part of MG’s central duct. This muscle is split from the orbicularis muscle. Also, [30] reported that, in addition to the contraction of Riolan’s muscle, constant synthesis and secretion of lipid lead to pushing their secretion slowly toward the orifice, and the normal blinking causes the expression and spread of the meibum over the ocular surface.

The holocrine acini of the MG at 1 month are filled with fully developed meibocytes and peripherally located basal cells. According to the MG, it is followed by a holocrine mode of secretion, that is reflected in its structure. The basal cells at the peripheral margin serve as proliferating progenitor cells that differentiate into the new meibocytes. The generation of the new meibocytes takes approximately 4 days in the rat [31]. These cells are replaced by the mitosis of the germinal basal cells [32]. Then, the differentiating meibocytes begin the production of the lipid droplets and accumulation in their cells. Toward the center of the acini, the meibocytes become mature, and the cells appear approximately filled with numerous lipid droplets that vary in shape, but still, the cells are vital due to the presence of the intact nucleus. The hyper-mature meibocytes are present in the center and close to the connecting ductules. The components of the whole cell form the oily secretory meibum [33], where cells appear to shrink with the pyknotic nucleus and disintegrate the cell membrane. This gradient in the meibocytes maturation is also supported by [34] in mice and [22, 35] in humans.

The function of the MG is secreting the meibum, which is liquid at a lid temperature delivered from the ducts to the lid margin in the form of Meibomian oil [32]. The principal role of the Meibomian oil is to decrease the evaporation of the tear from the periocular surface. Also, the Meibomian oil has antimicrobial action because it contains fatty acids complex with mucin. Additionally, Meibomian secretion may produce sexually attractive pheromones [36].

The TEM focused on the acini of the MG because they are like sebaceous glands and any abnormalities in the lipogenesis are responsible for the dysfunction states of the MG [35], and the TEM in this research supported the gradient maturation of the meibocytes. From observations, the basal cells are characterized by a high nuclear to cytoplasm ratio, and their cytoplasm contains numerous mitochondria and a rough endoplasmic reticulum that plays a role in the synthesis of the internal cell protein. In humans, the cytoplasm contains numerous keratin filament bundles [22]. The basal cells are attached to the basal lamina by hemidesmosomes, while the neighboring differentiated meibocytes by desmosomes agree with [28]. The basal cells do not have lipid droplets, while the differentiating cells are accompanied with cell enlargement and lipogenesis [37].

The differentiating meibocytes appear with a small nuclear to cytoplasm ratio, this reaches the difference in the number of organelles and presence of lipid vesicles, but no keratohyalin granules are identified. Mitochondria and smooth endoplasmic reticulum are found between the meibum vesicles, and these vesicles are different in shape and size depending on the degree of maturation of the meibocytes. The mature cells are characterized by a large lipid vesicle and few intracellular organelles, which agrees with [28].

The MG is like the sebaceous gland, and well established that its function is regulated via sex hormones, which androgen supports their function, and estrogen acts antagonistically. In this research, the androgen receptor protein is expressed in the epithelial cells of the meibocytes and within the basal and ductal cells than the mature cells due to the accumulation of the lipid vacuoles. While [35] recognized that the androgen is reactive with the differentiating meibocytes and the mature cells but reactive weakly in the basal and ductal cells. The different distribution and reactivity suggest that the androgen acts principally in the differentiating and mature cells to regulate the activity of this gland.

The same results were published by other researchers who reported that the MG is considered the androgen target organ and plays a role in regulating lipid and gene expression production in the gland [38]. They added that any deficiency in the androgen leads to dysfunction of the MG, especially in women who are associated with changes in the MG, an increase in the symptoms and signs of dry eye, and altered lipid patterns in the secretion of the MG [24, 39, 40]. Another clinical finding is demonstrated as painful eye, high sensitivity to light, blurred vision, orifice metaplasia, and a decrease in the tear film break uptime. The topical administration of androgen is an effective therapy and safe for MG diseases and patients with dry eye syndromes [32].

The MG is considered a large sebaceous gland, and the androgen controls the differentiation, development, and elaboration of the lipid in the sebaceous gland in nonocular sites [41]. These androgen receptors are believed to mediate their action in the nonocular tissue as the sebaceous gland [42]. They added that the deficiency of androgen altered by lipid production in the rabbit MG, but these changes are not necessary to be reflected externally on the ocular surface and contribute very little to the lipid layer of the tear film because, while it is a sharp contrast in humans that the MG is the primary source of the ocular lipid surface and the tear film, most lipids on ocular surface in the rabbit are produced from other adnexal tissues.

In this study on rabbits, the basal cell and the meibocytes with their nuclei reacted with the estrogen receptor antibodies, where increase in age increases the expression of the estrogen receptors. While in humans, [43] stated that no expression of estrogen changes is associated with gender or age.

The positive proportion of estrogen in the basal cells increased significantly with age [44]. This result agreed with our research, and this may be due to a decrease in the level of hormone in the blood of male and female with age, where the level of testosterone in the serum decreases with age, and this leads to estrogen upregulation. As a high concentration of estrogen is present locally compared to the low overall serum estrogen level, in men, the serum estrogen produces 20% from Leydig cells and 80% from the peripheral fat tissue, where the testosterone circulating is metabolized in the fibroblast, whereafter, the testosterone transforms to estrogen in the tissue, where estrogen is present. This explains why no differences in the density of the estrogen in the sex-related MG are found.

The tear breaks uptime depends on the lipid layer of the tear film; no proportion is found between the estrogen-positive cells and the fat layer of the tear film [44]. Estrogen effect on the MG has antagonized the action of androgen that led to suppression of lipid synthesis and promotion of the MG dysfunction and evaporative dry eye [45]. In contrast, the reduction in the action of androgen rather than an increase in estrogen action is responsible for the greater prevalence of dry eye [46].

Cytokeratin (CK14) is the marker for the basal and differentiating cells, which is present in the basal cells of stratified epithelium and the epidermis stem cells. These cells express CK14 as the primary keratins, and CK14 is downregulated during differentiation and converted to other cytokeratins [47]. Our investigation showed that the basal cells more highly reacted to CK14 than the differentiating and the mature cells. Also, the ductal epithelium expresses the positive reactions to CK14, and these different expressions are in accordance with the amount of lipid production inside the cells.

CK14 is expressed in the duct and acini of the MG, which is found in three to four basal layers and the ductal epithelium of MG near its orifice [14]. The duct of the MG is considered a major site for the epithelial stem cells that give rise to mucocutaneous junction posterior to the duct of the MG and epithelium of the palpebral conjunctiva. Also, the duct cells of the MG have self-renewal ability, and this is common in all stem cells [14]. The MG can transform into the hair follicle, which is clinical as atopic production of vibrissae (distichiasis). Therefore, the MG contains multipotent cells [48].

## Data Availability

The datasets generated and analyzed in the current study are available from the corresponding author upon reasonable request.
